# A 14-year girl with abdominal pain: case report of splenic cyst 

**Published:** 2019

**Authors:** Pantea Tajik, Amir Hossein Goudarzian, Zeinab Pourzahabi

**Affiliations:** 1 *Department of Pediatric Gastroenterohepatology, Amiralmomenin Hospital, Semnan, Iran*; 2 *Student Research Committee, Mazandaran University of Medical Sciences, Sari, Iran*; 3 *Department of Pediatrics, Amiralmomenin Hospital, Semnan, Iran*

**Keywords:** Children, Splenic cyst, Pain, Case report

## Abstract

The spleen cyst is a rare and often incidental finding that can be primary or secondary, parasitic or non-parasitic. Based on the size of the cyst, the patient may experience symptoms and plan of treatment based on size and symptoms will be done. Our patient was a 14 years old girl who had two years of vague abdominal pain and was referred to our hospital with severe abdominal pain. In the CT scan, three large cysts were observed in the spleen; another surgical consultation was done and recommendation for splenectomy was made. The patient became hydrated and treated with ceftriaxone and metronidazole.

## Introduction

 The spleen cyst is a rare disease that is commonly coincidental finding in the imaging, or as a result of physical examination of a patient with left upper quadrant pain, left shoulder pain, abdominal distention, or splenomegaly. It can be associated with many abnormal conditions including: Post-traumatic, Pseudocysts, cystic splenosis, Hydatid (echinococcal), Congenital, Epidermoid, Mesothelial cyst, Hemangioma, lymphangioma, Polycystic kidney disease with splenic cysts and cystic metastasis to the spleen (1-3).

Our case is about a patient with abdominal pain with splenic cyst which eventually treated with a diagnosis of splenic cyst. 

## Case Report

A 14 years old female patient who came to the emergency room due to abdominal pain located in the LLQ (Left Lower Quadrant) and RLQ (Right Lower Quadrant). According to the patient, she had a mild pain in the epigastric area since the night that she took her Ranitidin and her pain was resolved, but she had sudden and sever pain in the hypogastric area since the morning so she woke up from pain. And simultaneously have nausea and vomiting. Due to severe abdominal pain, she did not have urinary symptoms and had no excretion since the night before the visit. Her mother said that she has had vague abdominal pain about two years ago, but no specific paraclinical examination was performed.

The patient was admitted for the treatment of appendicitis with primary diagnosis of acute abdomen. In the initial examinations, clear tenderness in the area of RLQ, LLQ, LUQ (Left Upper Quadrant) of abdomen and rebound tenderness was positive the examinations of other organs were normal and there was no other positive finding. 

Experiments were conducted in which leukocytosis was observed, but urinalysis and urine culture tests were normal (table 1). An ultrasound scan was performed that showed no evidence of appendicitis, but in the spleen it was seen umbilical a cyst with an internal echo and septum, measuring 115 * 75 mm. Emergency surgical consultation was carried out. At the time of counseling, the pain of the patient was reduced and a slight tenderness was observed in the left side of abdomen, and the rebound tenderness was resolved and, according to surgical consultation, it was recommended that the CBC be repeated and CT scan be performed.

**Table 1:** Laboratory tests results

WBC: 12.5 (X1000/cumm)	BS: 90 (mg/dl)
Hct: 42.2 (%)	ESR: 5(m.m)
Plt: 169 (X1000/cumm)	CRP: Negative
BUN: 13 (mg/dl)	K: 3.5 (meq/l)
Cr: 0.6 (mg/dl)	Na: 138 (meq/l)

**Figure 1:**
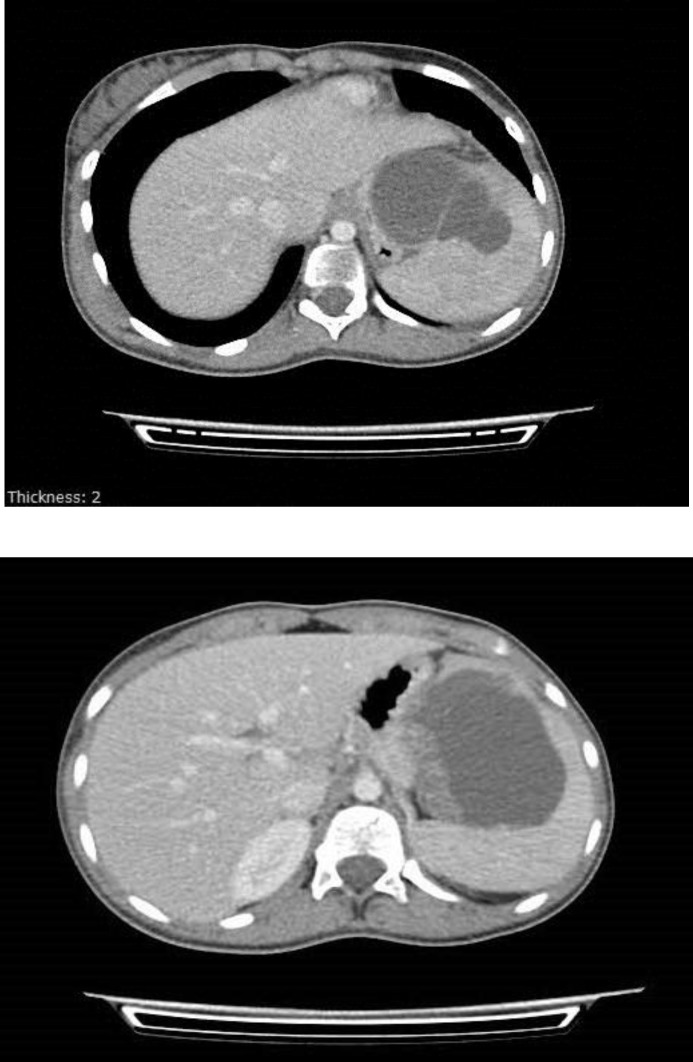
CT of the abdomen shows a splenic cyst

In the CT scan (figure 1), three large cysts were observed in the spleen; another surgical consultation was done and recommendation for splenectomy was made. The patient became hydrated and treated with ceftriaxone and metronidazole.

**Figure 2:**
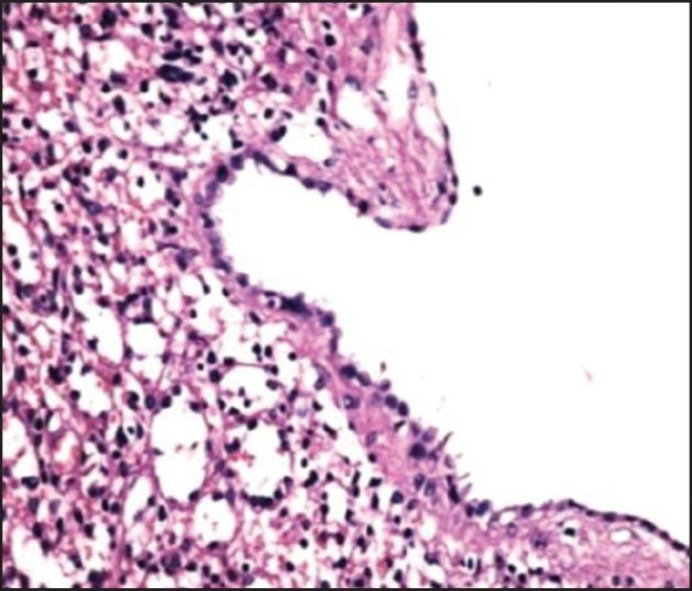
Histopathological examination of splenic specimen (epithelial cyst)

**Pathology report**

*Macroscopic*. Specimen received in fixative consists of a spleen measuring 15*12*7 cm. The capsular surface shows irregular and cystic. Cut surface shows multilacunar cyst. Inner layer was gray white and smooth.

Our patient was on the list of splenectomy surgery and received the necessary vaccines. After two weeks of vaccination, splenectomy was performed and the sample was sent for pathology. She is currently in good condition and does not have any complications. She now receives Penicillin V as a prophylactic antibiotic.

## Discussion

The cysts of spleen are rare lesions. The cause of the true cysts is unknown and there are different opinions on its pathogenesis. One idea is that they are formed from the mesothelium layer of the spleen surface during its growth (4). Patients have congenital splenic cysts are younger than those with false cysts, and the higher prevalence of true cysts in women has been observed (4, 5). The splenic cysts are divided into two groups parasitic or nonparasitic and based on an epithelial capsule can be classified as primary cysts (epithelial/true cysts), which have an epithelial capsule, or secondary cysts (pseudocyst /false) which have no capsule.

The secondary cyst can be formed due to trauma, abscess or necrosis (6-8). Splenic cyst may be epidermoid, transitional or mesothelial, or all three at once. Among splenic cysts lymphangioma is very rare and often subcapsular, having calcified mural lesions (9). Based on the size of the cyst, the patient may experience symptoms of abdominal pain, nausea, vomiting, thrombocytopenia, diarrhea and complications such as infection, rupture or hemorrhage (10, 11).

When cyst is less than five centimeters and asymptomatic, conservative treatment is recommended but if the cyst is larger and symptomatic, surgical treatment should be better (4, 12-16). 

Based on the size of the cyst and the probability of infection bleeding or rupture of the cyst the surgical indication is given to the patient and the aim is to reduce the tissue of the cyst and prevent its recurrence. There are several surgical methods for treating cysts, including percutaneous, open surgical, laparoscopic depending on the patient's condition (17). For our patient, total splenectomy was performed and the pathology of the cyst was epithelial cyst (figure 2).
